# Catching the bug: the influence of Thomas Henry on a young entomologist

**DOI:** 10.3897/zookeys.796.20761

**Published:** 2017-11-15

**Authors:** Katrina L. Menard

**Affiliations:** 1 Sam Noble Oklahoma Museum of Natural History, 2401 Chautauqua Ave., Norman, OK 73072 Sam Noble Oklahoma Museum of Natural History Oklahoma United States of America

When I was asked to write an appreciation of Tom Henry, I was a little bit overwhelmed. How can one person describe more than ten years of mentoring, collaboration, and friendship? Tom and I have worked together on several scientific papers, but this is the first time I have had to write about the influence he has had on my life.

My connection with Tom Henry started with luck. Growing up, I was always one of those nerdy kids who loved being outside and playing with nature. My special fondness was insects and other small creatures, which was noticed by my next-door neighbors in northern Virginia, both professional entomologists who worked with the Smithsonian. They suggested that if I wanted to know what it was like doing research on insects, I try volunteering at the Smithsonian Institution's Entomology Department in Washington, DC. At the time, I had no idea that entomology was a real job, and that museums were for more than just exhibits, so I emailed the Entomology Department via the website, asking if anyone wanted a young college-age volunteer.

Tom Henry was the first person to reply to my email that first summer before I started college. He wrote that he oversaw the Heteroptera collection (I had no idea what that was), and that he was part of the United States Department of Agriculture that worked at the Smithsonian. Other offers came from the Diptera collection, but since I have never liked maggots, I decided bugs were a better choice. Tom’s Collection Manager at the time, Michele Touchet, worked with me to get my paperwork started to volunteer at the Smithsonian.

Entering the Smithsonian with my newly printed badge and ready to start my first day, I wasn’t sure what to expect. My first thoughts were that maybe I was working for someone like Indiana Jones, who works in museums and keeps his stuff there but travels the world collecting little jeweled bugs. Or one of those crazy entomologists you see in your mind waving a net chasing anything that moves. In a way, both are true, but the man I met was a lot more real and tough. Tom came swinging through the big glass doors with his characteristic fast-paced walk and museum-work uniform of button-down shirt tucked into his jeans and open-toed sandals (always with socks), to pick me up to bring me to the collection with a bright smile and a hello. He was disarming in that he looked like a normal person, but had the charisma of someone I could relate to. Nervousness was quickly replaced by excitement, as I tried to keep up with him heading up to the collection. In many ways since that first day, I still feel it is almost impossible to keep up with Tom: his encyclopedic knowledge, his intense focus, and his work ethic are hard to beat, even for a ‘youngin’ like me.

That first summer working with Tom as a volunteer was pivotal in my life as an entomologist. He showed me that it was possible to open a whole other world of beauty and intrigue, if I took the opportunity to look, and that it was an exciting world. Every day that I volunteered or worked at the Smithsonian, no matter how tired Tom was from identifying the endless flow of “urgent identifications” that arrived on his desk, when I visited his office with a question or a specimen, he would always perk up at the opportunity to talk bugs. Sometimes, I would come with a question about a specimen just to hear him tell a story about it, such as his travels with Al Wheeler throughout the eastern United States, or climbing up a canopy tower in South America to collect odd bugs in the forest. He was always patient and accommodating, even when I probably was taking more of his time than he could spare.

Tom also encouraged me to grow as a professional entomologist. He and Michele taught me the basics of working in a museum and on collections while databasing nearly the entire Heteroptera collection, but Tom got me into bugs. Going through drawer after drawer, learning the names, the diversity, and being able to ask Tom questions about how he collected them, what they do, and why they are the way they are, led me on the path to becoming an expert. He fostered my curiosity of the group by having me sort bugs to family, then teaching me how to sort them to subfamily. Tom showed me the correct way to point-mount a bug, demonstrating from the constantly growing tower of glue on his desk how to do it the correct way. When I asked him a question he wasn’t sure about, he would do his characteristic eyebrow frown, swiftly turn from the research office, and find me an answer from his extensive reprint collection. There were not many questions he didn’t have answers to, since I have yet to meet someone else with such extensive knowledge of North American and South American bugs. He didn’t have to answer my questions, or give me as much personal attention, but he did, which showed me his kindness. We sometimes went out for lunch together to continue our talks about bugs outside of work, either getting sushi at the old All You Can Eat place that used to be in Rosslyn or meeting his wife Katy nearby at the Sculpture Garden, where he could also slyly tap the plants nearby looking for tingids.

Collecting bugs is Tom Henry’s forte, and I learned much from him on how to collect. It wasn’t until graduate school that I had the chance to be in the field with him, but Tom taught me all the tricks and tips on how to collect bugs. Seeing him in action is inspiring. In 2006, we all converged on Switzerland for a Plant Bug Inventory Planetary Biodiversity Inventory meeting, and did a short collecting trip in the Swiss Alps. All the bug stars were there: Gerry Cassis, Christiane Weirauch, Toby Schuh, Michael Schwartz, Tomohide Yasunaga, Denise Wyniger, and Tom Henry. With their nets out, it was all-out war who could catch the most and with the highest diversity. Everybody spread out and took out their best tricks of the trade, and I watched and learned. Ultimately, Tom got the most specimens and the highest diversity, winning the mirid scavenger hunt of the trip. Later, everyone celebrated their catches and shared stories of bug collecting over drinks and, for Tom and Tomohide, cigars.

After working at the Smithsonian, I maintained contact with Tom throughout my graduate school years, sending him specimens from Texas while at Texas A&M for my masters, and later when I joined the Plant Bug Planetary Biodiversity Inventory as a PhD student. We would sometimes visit the Smithsonian to help sort and catalog, and I would catch up with Tom and Katy. He is always a gracious host, and he and Katy showed their grace and thoughtfulness by hosting the International Heteropterist Symposium at the Smithsonian in 2014. It was a tremendous meeting, and he showed all of us how to not only be expert scientists, but expert hosts for colleagues from all over the world.

My hope is to continue to work with Tom on projects for years to come, and to keep picking his brain for knowledge and expertise. To me, Tom is irreplaceable, and it fills me both with happiness that as he approaches retirement he can spend less time on urgent requests and paperwork and more time on bugs, but also sadness, since I feel as if I have only just started to fully appreciate all his accomplishments as a professional scientist. I appreciate every ounce of knowledge and mentoring he has had for me, and treasure having him be a part of my life.

